# Microsphere Peptide-Based Immunoassay for the Detection of Recombinant Bovine Somatotropin in Injection Preparations

**DOI:** 10.3390/bios12030138

**Published:** 2022-02-22

**Authors:** Nathalie G. E. Smits, Toine F. H. Bovee, Sidharam P. Pujari, Leendert A. van Ginkel, Michel W. F. Nielen, Bauke Albada

**Affiliations:** 1 Wageningen Food Safety Research, Wageningen University and Research, 6700 AE Wageningen, The Netherlands; toine.bovee@wur.nl (T.F.H.B.); leen.vanginkel@wur.nl (L.A.v.G.); michel.nielen@wur.nl (M.W.F.N.); 2 Laboratory of Organic Chemistry, Wageningen University, Stippeneng 4, 6708 WE Wageningen, The Netherlands; sidharam.pujari@wur.nl

**Keywords:** epitope mapping, recombinant bovine somatotropin, peptide-based immunoassay, suspension array, biorecognition

## Abstract

The use of peptides in immunoassays can be favored over the use of the full protein when more cost effective or less toxic approaches are needed, or when access to the full protein is lacking. Due to restricted access to recombinant bovine somatotropin (rbST), a protein enhancing growth and lactating performances of livestock, which use has been banned in the EU, Canada and Australia (amongst others), we developed a peptide-based biorecognition assay on an imaging planar array analyzer. For this, we identified the rbST epitope that is responsible for binding to the rbST-targeting monoclonal antibody 4H12 (MAb 4H12) to be _115_DLEEGILALMR_125_. This linear peptide was synthesized and coupled to microspheres, after which it was tested in a biorecognition competitive inhibition assay format. We observed IC_50_ values of approximately 0.11 μg mL^−^^1^, which are lower than observed for the full rbST protein (IC_50_ = 0.20 μg mL^−1^). Importantly, there was no binding with the scrambled peptide. Preliminary results of directly coupled peptides in a microsphere biorecognition assay for detection of rbST are presented. Real-life applicability for detection of somatotropins (STs) in injection preparations of bovine-, porcine- and equine ST are shown. This newly developed immunoassay strongly supports future developments of peptide-based immunoassays to circumvent the limited access to the full protein.

## 1. Introduction

When immunoassays detect a full protein, the use of this full protein in assay building is commonplace. However, when access to the full protein is limited as shown in this study, where the regulatory ban in Europe and other countries limits access to recombinant bovine somatotropin (rbST), the use of protein-derived peptides offers advantages. In general, the use of peptides offers advantages in (i) cost effectiveness, (ii) toxicity, (iii) pathogenicity, (iv) specificity, and (v) limited access to the full protein. For instance, in the development of serological tests for COVID-19, the high costs for the S1-protein could be avoided using a 12-mer peptide, while at the same time showing increased specificity, resulting in an assay with high diagnostic performance [1]. In development of neutralizing antibodies for toxin therapy of, e.g., venoms, bacteria toxins, etc., antigenic peptides are used instead of the toxic full protein [2,3,4]. Antigenic peptides can also be used in peptide-based detection systems, for example, to avoid the use of infectious viruses and toxins as antigen in an immunoassay [5,6,7]. In diagnosing food allergy, peptide-based assays provide insights on epitope-specific antibody binding [8,9], which is related to pathogenesis, its prognosis and resulting treatment options of the disease [10]. In this study, we show the specific advantage of peptides when access to the full protein rbST is limited.

RbST is a 191 amino acid long protein hormone that enhances growth and lactating performance in cattle. When administered to dairy cows according to the manufacturer’s protocol, milk production can increase up to 25% [11]. The use of rbST for milk-enhancing properties is approved in several countries, but is banned in the European Union [12]. To control this ban, a strategy to detect rbST misuse in the EU has been described by Smits et al. [13]. The strategy for rbST in serum and milk from dairy cows is based on the digestion of the proteins and detection of rbST-specific peptides formed using an extensive dedicated LC-MS/MS method. To distinguish between farms suspected and not-suspected of rbST misuse, a straightforward immunochemical method measuring rbST induced-antibodies can be performed as an initial screening procedure. To measure the rbST-induced antibody expression, the full rbST protein is attached to a variety of surfaces, e.g., microspheres for suspension arrays [14,15], 96-well plates in ELISA [16,17] and glass chips in microarrays [18], enabling detection by binding to the immobilized rbST [19]. As these assays rely on the banned full rbST protein, they suffer from limited accessibility and applicability. Moreover, promising immunoassays for rbST detection itself, use the temporary available polyclonal antibodies [20,21], and others only demonstrated rbST detection in buffer [22] or show antibodies lacking specificity [23]. To avoid the dependence on the full protein, or an antibody source which can be exhausted, we developed an immunoassay that is built on a specific peptide part to represent the full protein and a monoclonal antibody, which can be produced indefinitely.

First, we performed an epitope mapping of rbST using both linear and conformational epitope mimics [24] in an ELISA-based format [25]. After identification of suitable peptide epitopes, we synthesized the relevant peptides, and scrambled versions thereof, and covalently immobilized them to a carboxyl-rich surface of microspheres using an EDC/NHS protocol [26,27,28,29,30]. This enabled us to develop a suspension-based format that uses immobilized peptides for the detection of rbST.

## 2. Materials and Methods

### 2.1. Materials

The rbST protein standard was a gift from the National Hormone & Peptide Program (NHPP) of Dr. Parlow (Torrance, CA, USA) and equine somatotropin (eST) was from Bresagen (Adelaide, Australia). We obtained Posilac^®^ (methionine-rbST) sometribove zinc suspension for injection, and excipient (sesame oil and aluminum monostearate) for injection from Elanco (Indianapolis, IN, USA), and Hilac^®^ (alanine-rbST) was obtained from LG (Seoul, Korea). The hybridoma cell line producing monoclonal antibody (MAb) 4H12 (IgG2b, κ) was developed and produced by Genscript (Leiden, The Netherlands). Chemicals for peptide synthesis were obtained from the following commercial sources: 1-hydroxybenzotriazole hydrate from Acros (Geel, Belgium); PyBOP, Fmoc-Gly-OH, Fmoc-Ile-OH, Fmoc-Ala-OH, Fmoc-Met-OH, Fmoc Arg(Pbf)-OH, Fmoc Asp(O*t*Bu)-OH, Fmoc-Leu-OH, Fmoc-Glu(O*t*Bu)-OH, Fmoc-Gly-OH and Rink Amide MBHA resin HL were purchased from Novabiochem (Zwijndrecht, The Netherlands); HBTU, dichloromethane, *N,N*-dimethylformamide and 2-propanol from BioSolve (Valkenswaard, The Netherlands). Acetic anhydride, trichloroacetic acid, sodium dihydrogen phosphate monohydrate, sodium chloride, sodium hydroxide, sodium azide, tween-20 and hydrochloric acid were supplied by Merck (Zwijndrecht, The Netherlands). Porcine Somatotropin (pST), human Somatotropin (hST), bovine serum albumin (BSA), 2-(*N*-morpholino) ethanesulfonic acid (MES hydrate), sodium phosphate dibasic, boric acid, *N*-hydroxysulfosuccinimide sodium salt (sulfo-NHS) and *N*-(3-methylaminopropyl)-*N*-ethylcarbodiimide (EDC) were purchased from Sigma-Aldrich (Zwijndrecht, The Netherlands). Trifluoracetic acid and *N,N*-diisopropylethylamine were purchased from Fisher Scientific (Landsmeer, The Netherlands). The analytical LC column ZORBAX Eclipse XDB-C18, the preparative column PreptHT XDB-C18, 21.2 mm I.D. × 250 mm, the Agilent model 1260 Infinity II LC system and the LC/MSD were all supplied by Agilent (Amstelveen, The Netherlands). Triisopropylsilane and ninhydrin were obtained from TCI (Zwijndrecht, Belgium) and Fmoc-ε-Aca-OH and Boc-mini-PEG-OH from Peptides International (Louisville, KY, USA). Drive fluid, paramagnetic color-coded microsphere sets 012, 043, 038, 029, 073 and 064 and the planar microsphere array analyzer (MAGPIX) running on XPONENT software were from Luminex (Austin, TX, USA) and Phycoerythrin (PE)-labeled goat anti-mouse immunoglobulins (GAM-PE) were from Moss (Pasadena, MD, USA). Protein Lobind Tubes were supplied by Eppendorf (Hamburg, Germany) and the Snijder test tube rotator was from Omnilabo International (Breda, The Netherlands). The microtiter vari-shaker was purchased from Dynatech (Guernsey, UK) and the magnetic particle concentrator DynaMag™-2 was from Invitrogen Dynal (Oslo, Norway). The ultrasonic cleaner was obtained from VWR International (Amsterdam, The Netherlands) and the centrifuge was from Hermle (Wehingen, Germany). The DS-11 series spectrophotometer was from Denovix (Wilmington, DE, USA).

### 2.2. Epitope Mapping

**Figure 1:**
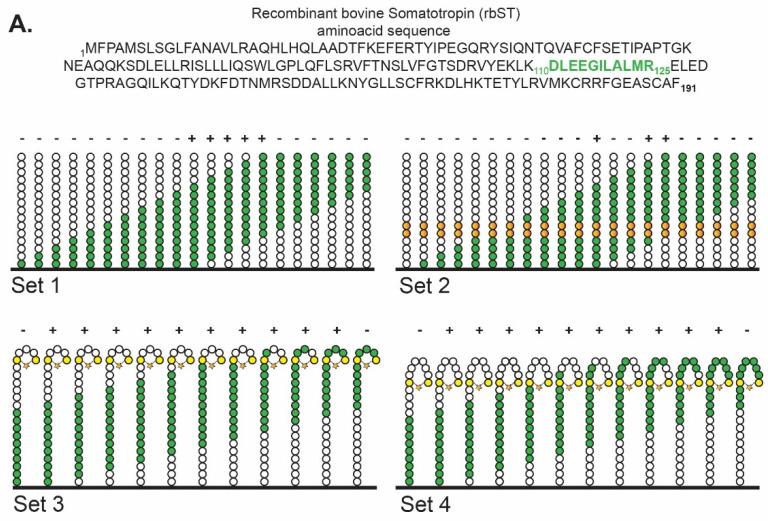

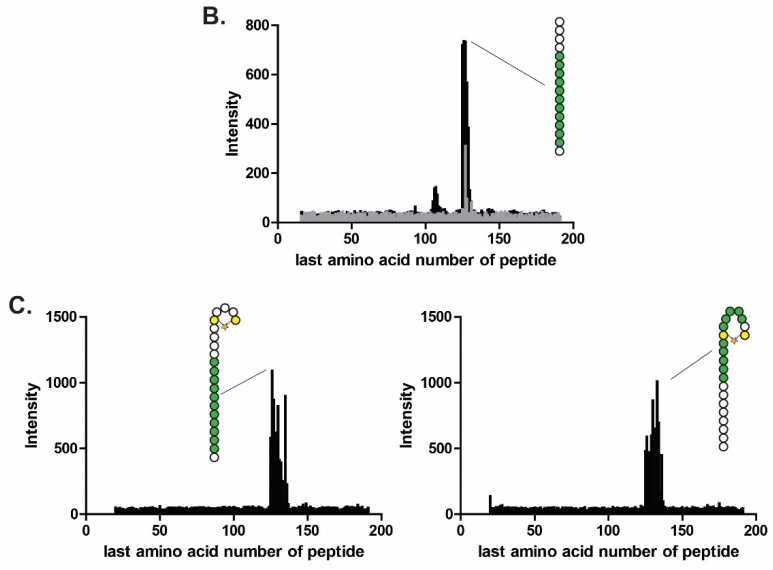
Schematic representation of the four epitope mapping approaches to identify peptide-based mimics of recombinant bovine somatotropin (rbST) that bind to the monoclonal antibody 4H12. (**A**) The entire rbST amino acid sequence is shown at the top. In the schematic representation of the peptides, only a smaller number of peptides, including the binding epitope, is shown for clarity purposes. Set 1 represents linear peptides of 15 aa with an offset of 1 aa in each subsequent peptide; set 2 represents the same linear peptides as used in set 1 with the 10th and 11th amino acids replaced by alanine or glycine (in case a native alanine is present) (orange beads); set 3 represents peptides of 21 aa with an offset of 1 aa that mimic an α-helical loop with the first and fifth aa replaced by cystine residues (yellow beads), which are connected to an MP2 clip (star); set 4 is similar to set 3, with this exception that the cysteine amino acids are placed on the first and eighth aa. The binding epitope is presented in green in both the sequence of rbST and, schematically, in the four sets. The presence of a potential binding epitope for the antibody is schematically presented by a ‘+’, and absence of binding is presented by a ‘-’. (**B**) Intensity results for epitope mapping of monoclonal antibody 4H12 for the rbST protein sequence; binding of antibody 4H12 to 15-mer linear peptides (set 1, black) and peptides subjected to a double-Ala scan (set 2, grey). (**C**) binding of antibody 4H12 to 21-mer sequences that contain short and extended α-helical turn mimics (set 3, left graph, and set 4, right graph). The highest intensity is schematically represented in the respective graph, the green beads in the schematic depiction represents the binding epitope.

The entire sequence of rbST (Figure 1A) was epitope mapped for antibody 4H12 by Pepscan Presto BV (Lelystad, The Netherlands), as described before [31]. Briefly, the rbST sequence was derived from the endogenous bST sequence by replacing the N-terminal alanine (Ala) with a methionine (Met) residue. With this rbST sequence, four sets of peptides were produced and used to determine the binding epitope of MAb 4H12: (i) linear peptides of 15 amino acids in length with an offset of one amino acid (set 1), (ii) linear peptides similar to set 1, with the amino acids on place 10 and 11 replaced by alanine (Ala); a native Ala present on place 10 and/or 11 was replaced by glycine (Gly) (set 2), (iii) α-helical loop mimics of 21 amino acids in length with an offset of one amino acid and cysteine residues on place 1 and 5 to enable connection with an α-helix-inducing mP2 chemical linkage of peptides onto scaffolds (CLIPS, set 3) and (iv) α-helical loop mimics similar to set 3, with the two Cys residues placed on amino acid numbers 1 and 8, which were also linked with mP2 CLIPS (set 4). Binding of Mab 4H12 to the described peptides is quantified using an ELISA-type read out.

### 2.3. Peptide Synthesis

**Figure 2:**
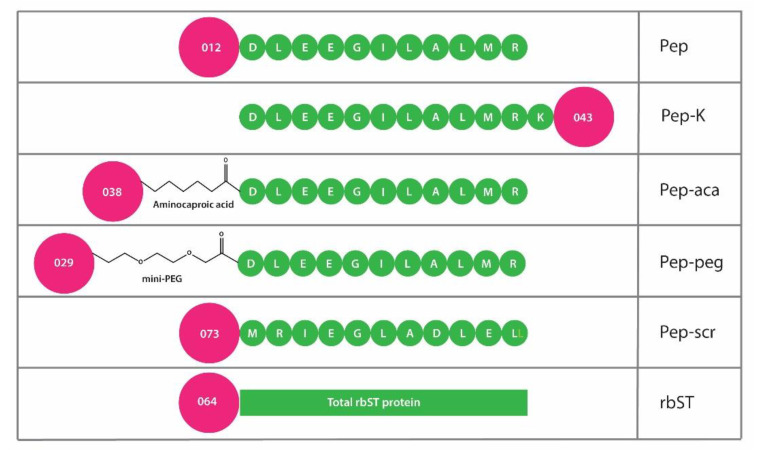
Schematic overview of the magnetic microspheres with their respective microsphere numbers (pink beads). Individual amino acids are presented as a green bead and single letter amino acid code. Abbreviations of the coupled peptide or protein as used in this manuscript are mentioned next to the schematic presentation; linear peptide (pep), linear peptide with extra lysine (pep-K); linear peptide with peg spacer (pep-peg); linear peptide with aca spacer (pep-aca); linear peptide scrambled (pep-scr); total recombinant bovine somatotropin protein (rbST).

Three peptides from the epitope mapping were selected and synthesized: DLEEGILALMR (pep), DLEEGILALMRK (pep-K) and MRIEGLADLEL (pep-scr) (Figure 2). For this, 1 mmol of the Fmoc-protected amino acids were weighted separately and placed in the synthesizer. Peptides were built on 0.25 g of Rink Amide resin (loading capacity of 0.6–1 g/mmol, 3:1 amino acids:resin) by standard Fmoc synthesis. To start, the resin was pre-swelled for 3 min in DMF. For Fmoc-removal, the resin was washed two times for 30 s with DMF followed by incubating 1 × 5 min and 1 × 20 min with 20% piperidine in DMF. The piperidine solution was removed by washing for 2 × 30 s with DMF, 1 × 30 s with DCM and 3 × 30 s with DMF again. The next Fmoc-protected amino acid was activated by 2.5 mL 0.4 M HBTU and 2.5 mL 0.8 M DIPEA in DMF for 3 min, which was then added to the resin and incubated for 90 min. This procedure was repeated until the desired peptide sequence was generated. When desired, the peptide was functionalized in the last step before cleavage from the solid support. Specifically, pep-K was acetylated on the N-terminus using 4.7 mL Ac_2_O, 230 mg HOBT•H_2_O and 2.2 mL DPEA in 100 mL DMF, followed by washing with DMF and DCM (each three times for 2 min, with 2 mL of solvent). For the peptides that contained a spacer, the Fmoc-group was removed and the spacer was coupled to the N-terminus using the coupling procedure mentioned above, but now with Boc-protected amino caproic acid (aca) or Boc-protected mini-PEG-COOH (peg). After modification, peptides were disconnected from the resin by incubation with 2 mL 95% TFA, 2.5% triisopropylsilane and 2.5% water (4 h). Each peptide was collected in a tube and precipitated by the addition of diethylether (−20 °C) and centrifuged for 5 min at 3800 g. Then, the supernatant was removed and the pelleted peptide was dried by air at room temperature in the fume hood.

### 2.4. Peptide Purification

Peptide purification was performed using a preparative LC system connected to a single quadrupole mass spectrometer (MS) for mass confirmation of the synthesized peptides. A total of 500 µL from the prepared peptide solution was injected onto the preparative column. The flow rate was set at 20 mL min^−1^ and 0.1% formic acid was added to the mobile phases. A gradient was used starting with 90:10 (*v/v*) water/acetonitrile, increasing to 50:50 (*v/v*) water/acetonitrile in 14 min. This mobile phase composition was kept constant for 3 min. The next minute the gradient was returned to starting conditions of 90:10 (*v/v*) water/acetonitrile and this was kept constant for 7 min. Total run time was 25 min. The MS was operated in positive electrospray ionization mode. Masses of synthesized and modified peptides of interest, purified by HPLC, were verified by MS and fractions containing peptides were collected manually. [M+2H]^2+^ ions at *m/z* 630, *m/z* 715, *m/z* 702.5, *m/z* 686.5 and *m/z* 630 were found for, respectively, pep, pep-K, pep-peg, pep-aca and the scrambled peptide pep-scr, in accordance with the expected theoretical values (Supplementary Materials, Figure S1). After purification and fraction collection, the synthesized peptides were freeze dried for further use.

### 2.5. Microsphere Preparation for Microsphere Peptide-Based Immunoassays (MIPA)

Microsphere preparation was executed according to Bremer et al. [32] with the exception that Magplex^®^ microsphere sets instead of seroMAP microspheres were used. During the procedure, microspheres were trapped using a magnet (1 min) and after removal of the supernatant, trapped microspheres were resuspended by vortexing (1 min). The peptides pep, pep-K, pep-peg, pep-aca and rbST standard were coupled to the different internally dyed microsphere sets, i.e., microsphere set numbers 012, 043, 029, 038 and 064, respectively (Figure 2). The internal dye gives the microsphere set a unique spectral signature, and consequently, a unique read-out region. For each microsphere set, 2.5 × 10^6^ microspheres were covalently coupled with a two-step carbodiimide reaction using 500 μL of 100 µg mL^−1^ peptide in boric acid solution (100 mM boric acid, 1M NaCl, adjusted to pH 8.3 with NaOH), and 100 µg mL^−1^ rbST in MES buffer (50 mM MES pH 5.0). After coupling, the microspheres were stable for over 1 year when stored in a blocking buffer (PBS, 0.1% BSA, 0.02% Tween-20 and 0.05% NaN_3_) at 2–8 °C in the dark until use, with the exception for the microspheres measured with XPS, which were stored in Milli-Q water until use.

### 2.6. Inhibition MIPA Procedure

**Figure 3:**
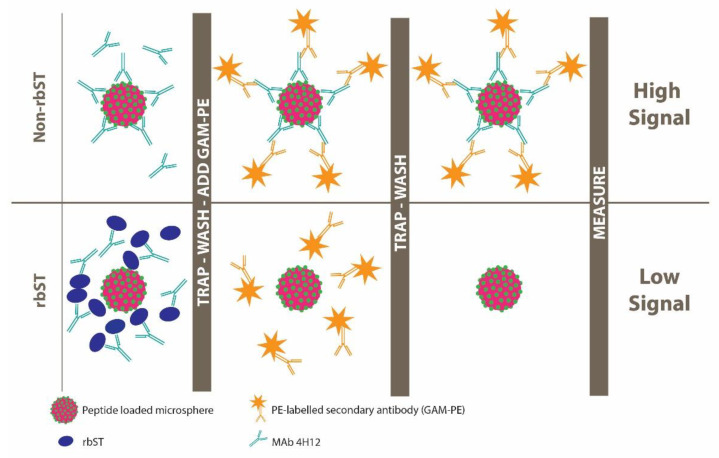
Summarized workflow and assay principle of the indirect competitive assay format for somatotropins. In absence of rbST (upper part), MAb 4H12 binds to the peptide-coated microspheres, which enables binding of the PE coupled secondary antibody. The presence of PE results in a high signal in the imaging planar array analyzer. In presence of rbST (lower part), MAb 4H12 binds to the rbST in solution. This results in no binding of MAb 4H12 to the peptide-coated microspheres, and therefore, no binding of PE coupled secondary antibody. As no PE is present, this will result in a low signal in the imaging planar array analyzer.

In this procedure, all dilution and washing steps were executed using PBST containing 0.1% BSA. In total, 100 μL of rbST, eST, pST and hST standard solution or injection preparation extract were added to a low bind 96-well microtiter plate. Next, 10 μL of 20-, 2-, 2-, 1- and 0.7 ng mL^−1^ antibody for microspheres coated with, respectively, pep, pep-peg, pep-aca, k-pep and rbST and microspheres (10 μL diluted suspension containing about 1250 microspheres per microsphere set) were added to each well. The microtiter plate was incubated for 20 min on a microtiter plate shaker to allow competition between the immobilized rbST epitope peptides or protein on the microspheres and the somatotropin (ST) present in the solution for the available antibody binding sites. After incubation, the microspheres were trapped by a magnet and washed twice. After washing, a 125 μL, 625-times diluted PE labeled Goat anti Mouse antibody was added and incubated for 20 min on a microtiter plate shaker. After this incubation step, microspheres were trapped with a magnet, supernatant removed and 125 µL PBST containing 0.1% BSA added. The plate was briefly mixed on the microtiter plate shaker before measuring on the imaging planar array analyzer (MAGPIX). The MIPA procedure for the detection of somatotropins is summarized in Figure 3.

### 2.7. Extraction of rbSTs of Injection Preparations

Proteins were extracted from syringes containing methionine-rbST, alanine-rbST and an excipient according to Heutmekers et al. [20]. Briefly, 5 mL CAPS buffer (50 mM CAPS, 100 mM NaCl pH11) was added to 100 mg injection preparation and the mixture was vortexed (1 min), sonicated (10 min) and centrifuged for 10 min at 2000× *g*. The buffer layer was separated from the white layer on top (slow-release formula) and filtered through a 5 µm filter. Protein concentrations were measured with a spectrophotometer.

### 2.8. X-ray Photoelectron Spectroscopy (XPS)

Prior to XPS analysis, modified microspheres (in Milli-Q) were dropcasted on a plasma-cleaned piece of gold and dried in a vacuum oven at 50 °C for at least 2 h. Microspheres and modified gold surfaces were analyzed using a JPS-9200 photoelectron spectrometer (JEOL Ltd., Tokyo, Japan). The spectra were obtained using monochromatic Al Kα Xray radiation at 12 kV and 20 mA with an analyzer energy pass of 10 eV for narrow scans. The obtained spectra were processed using the CASA XPS peak fit program (Casa Software Ltd., version 2.3.16 PR 1.6).

## 3. Results and Discussion

### 3.1. Identification of Epitopes Recognized by MAb 4H12 in rbST

To determine which peptide could mimic the rbST epitope that is recognized by the MAb 4H12 the Pepscan technology for epitope-mapping was used with four different peptide designs (Figure 1). For all four epitope designs, binding between MAb 4H12 and the sequence _115_DLEEGILALMR_125_ was demonstrated (Figure 1B). Especially, in the library containing 15-mer linear peptides of the full rbST protein (set 1), binding to the linear sequence _115_DLEEGILALMR_125_ was found, which was confirmed by a library containing double alanine replacements (set 2), showing the importance of amino acids _120_ILALMR_125_, with the highest negative impact when L_121_ and M_124_ were part of the replacement by alanine with a measured intensity reduction of more than 92% and 83%, respectively. These findings were confirmed by two other libraries (sets 3 and 4), which contained short and extended α-helical loop mimics of the peptide sequences that covered the entire primary sequence of rbST (Figure 1C). Therefore, we synthesized and used the straightforward linear peptide _115_DLEEGILALMR_125_ that was able to bind the MAb 4H12 for our further immunoassay development. We tested whether attachment to the microsphere via either the C- or the N-terminus of the peptide was preferred (the former option was facilitated by a C-terminally positioned Lys residue), and whether the biorecognition would benefit from the presence of a hydrophilic or hydrophobic spacer between the peptide and the microspheres (Figure 2).

### 3.2. Performance of the Microsphere Peptide-Based Immunoassay (MIPA)

**Figure 4:**
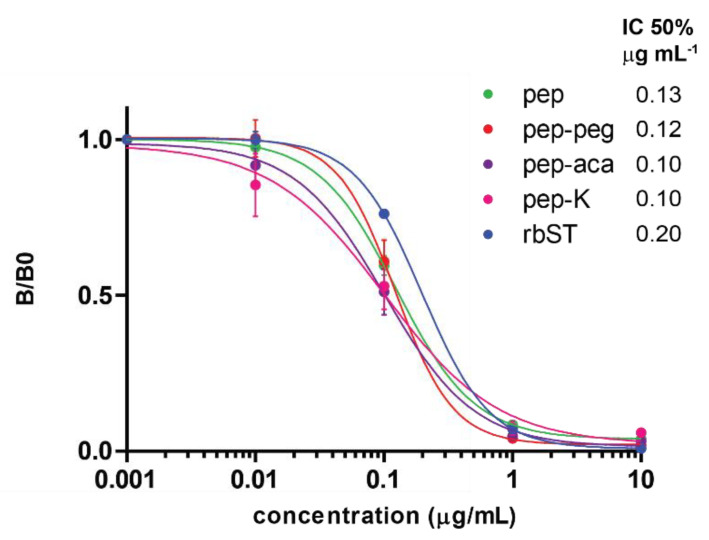
Typical inhibition curves, four-parameter fitted, of rbST in PBST/BSA solution for microspheres coupled with pep (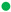), pep-K (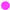), pep-peg (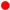), pep-aca (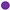) and the total rbST protein (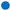) (*n* = 3). Error bars display the standard deviations on B/B0 per tested rbST concentration for each individual microsphere set. The half maximal inhibitory concentration (IC_50_) for each individual microsphere set is shown in the top right corner of the graph. Pep-scr did not show any binding of the MAb; consequently, its inhibition could not be included in this figure.

MAb 4H12 showed binding with all microsphere sets, i.e., to full rbST protein and to the mimicking epitope _115_DLEEGILALMR_125_, irrespective of the method by which it was attached to the surface. For example, attachment via the C-terminal additional Lys residue or the backbone amine group of the N-terminus yielded the same results with IC_50_ values of 0.10 and 0.13 µg mL^−1^, respectively. Similarly, IC_50_ values of 0.10 and 0.12 µg mL^−1^ were obtained for peptides attached to the surface via the hydrophobic aminocaproic acid (aca) or hydrophilic mini-PEG (peg) linker, respectively, which was positioned on the N-terminus, indicating that an additional spacer was not required (Figure 4).

Although the literature often describes the need for spacers in peptide-based biorecognition assays [33,34,35], in our assay format based on the MAb 4H12 and the peptide _115_DLEEGILALMR_125_ biorecognition pair, no spacers were required. As expected, no binding occurred of the mAb to the scrambled peptide coupled to a microsphere, showing specificity of MAb 4H12 for the peptide _115_DLEEGILALMR_125_. The presence of peptides on the microspheres was proven by XPS measurements (Supplementary Materials, Figure S2). In addition, the specificity was compared by initial surface plasmon resonance (SPR) experiments, where the pep-peg peptide was coupled to one microfluidic channel and rbST to another channel of the sensor surface. The sensor channels were incubated with two different rbST-induced monoclonal antibodies, MAb 4H12, which specifically binds the peptide _115_DLEEGILALMR_125_ and MAb 5E2, which recognizes another, yet unknown, epitope. Both MAbs showed binding to the immobilized rbST, as expected, but only MAb 4H12 was able to bind to the immobilized pep-peg, independently confirming its specificity (Supplementary Materials, Figure S3 and Table S1). Next, MAb 4H12 concentrations were optimized for the microsphere peptide-based immunoassay to a dilution factor corresponding to a signal intensity of 1000 MFI for the blank standard (dilution buffer, Supplementary Materials, Table S2). This resulted in MAb 4H12 concentrations of 20, 2, 2, 1 and 0.7 ng mL^−1^ for microspheres coated with pep, pep-peg, pep-aca, pep-K and rbST, respectively. The antibody concentrations in combination with the respective microspheres were used to determine the sensitivity of MAb 4H12 for the determination of full rbST-protein in standard solutions, as shown in Figure 4. For bot, rbST-protein and peptide immobilized microspheres, typical dose-response curves were obtained, with IC_50_ values of, respectively, 0.20 µg mL^−1^ and 0.11 µg mL^−1^. This shows use of peptide-immobilized microspheres give at least comparable sensitivities to rbST immobilized microspheres, although generally, affinity for the full protein is higher than the affinity for the single peptide. Further experiments were executed using microspheres coupled with pep, as all peptides showed similar results and pep is the cheapest and most straight forward peptide to obtain.

### 3.3. Application of MIPA to Somatotropin Injection Samples

Final protein concentrations in Posilac^®^, Hilac^®^ and excipient extracts were 5, 3.5 and 17 mg mL^−1^ respectively, in accordance with previously described extraction efficiency [20]. Although Posilac^®^ and Hilac^®^ contain a slightly different form of rbST—as the amino acid sequence of Hilac^®^ is similar to the native bST, whereas in Posilac^®^ the N-terminal amino acid alanine is replaced by a methionine—the MAb 4H12 binding epitope in both rbST forms is intact. Comparison of the injection sample extracts with the rbST protein standard showed 100% cross reactivity in the MIPA and was demonstrated on all peptide microsphere sets, whereas the excipient showed no cross reactivity.

**Figure 5:**
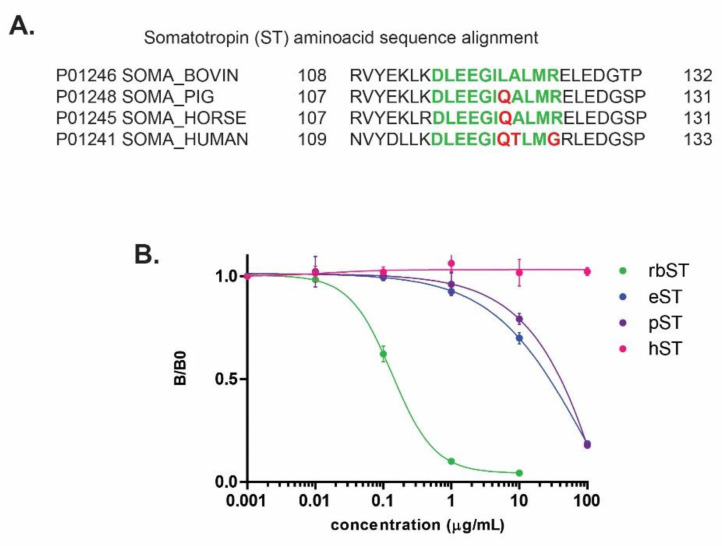
Amino acid sequence alignment for the epitope recognized by Mab 4H12 (green) and its variations (red) for bovine, porcine, horse and human Somatotropin (**A**). Inhibition curves of rbST (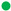), porcine somatotropin (pST, 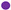), equine somatotropin (eST, 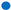) and human somatotropin (hST, 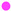) (*n* = 2) in PBST/BSA solution for microspheres coupled with pep (**B**).

To test the applicability of the newly developed assay for ST detection from other species, which would be an advantage for enforcement purposes, Uniprot sequences of other STs were compared for the MAb 4H12 binding epitope and showed only small varieties (Figure 5A). For pST and eST, only one amino acid was different compared to the rbST epitope identified by our epitope mapping study: one of the two amino acids with the highest impact for MAb 4H12 binding, L_121_, is replaced by Q. For hST, three epitope-related amino acids were different: L_121_, A_122_ and R_125_ were replaced by Q, T and G, respectively (Figure 5A). Calibration curves of pST, eST and hST showed that due to the replacement of three amino acids in hST, MAb 4H12 was not able to recognize and bind to hST, even at the highest tested concentration of 100 µg mL^−1^ (Figure 5B). However, cross-reactivity was seen for eST and pST, in which L_121_ is exchanged by Q. As earlier described, a signal reduction of 90% is expected when L_121_ is replaced, which is clearly demonstrated in the calibration curve at 1 µg mL^−1^ when the inhibition of eST and pST is compared to the inhibition of rbST. Here, rbST almost completely inhibits MAb 4H12, whereas inhibition by eST and rbST just started (Figure 5B). Full inhibition by eST and pST is observed around 100 µg mL^−1^. These results show specificity of MAb 4H12 for the peptide and demonstrates the possibility to make an estimation on cross-reactivity, and applicability of the MIPA for other ST at forehand, instead of empirically as done in Heutmeker et al. [20]. Moreover, Heutmekers et al. [29] used polyclonal antibodies in combination with the full rbST protein, of which there is limited access or, for the former, will be exhausted at one point. Both sources of the developed MIPA, MAb 4H12 and peptides, can be produced endlessly, so this MIPA can always be produced and used. Therefore, this MIPA is applicable for screening purposes of injection preparations containing rbST, pST and eST, and a simple 500-fold dilution of injection preparation extracts should be sufficient to detect these somatotropins, as injection preparations typically contain hundreds of milligrams somatotropins per injection. Furthermore, this approach enables us to ‘flag’ suspect samples, which would be an advantage for enforcement purposes, provided a confirmatory method is available, which is the case.

## 4. Conclusions

Compared to the full protein rbST MIPA, the peptide-based MIPA was found to be more sensitive with IC_50_ of 0.20 and 0.11 μg mL^−1^, respectively. The epitope recognized by MAb 4H12 was determined as DLEEGILALMR, and optimal results were obtained in MIPA when this peptide was attached to the N-terminus without any spacers. A new coupling procedure enabled direct coupling of the peptide to the microspheres and its successful attachment was confirmed by XPS. Real-life applicability was demonstrated for injection preparations of rbST, eST and pST. It is, therefore, important that we present an approach that can circumvent the denied access to the full rbST protein, and opens a new research field for future assay development for the detection of rbST.

## Data Availability

The data presented in this study are available on request from the corresponding author.

## Supplementary Materials

The following supporting information can be downloaded at: https://www.mdpi.com/article/10.3390/bios12030138/s1, Figure S1: HPLC-DAD chromatograms of peptide purification; Figure S2: Narrow scan XPS spectra for microspheres; Figure S3: Surface plasmon resonance analysis of Mab 4H12 with rbST and pep-peg; Table S1: Surface plasmon resonance response units of Mab 4H12 with rbST and pep-peg response units; Table S2: Mean fluorescence intensities of Mab 4H12 with peptide and rbST coupled microspheres.
