# Supplementary material for: Microsphere Peptide-Based Immunoassay for the Detection of Recombinant Bovine Somatotropin in Injection Preparations
Source: Biosensors (Basel). 2022 Feb 22;12(3):138. doi: 10.3390/bios12030138 (PMC8946150; doi:10.3390/bios12030138)
Supplement: Supplementary file 1 [file biosensors-12-00138-s001.zip › biosensors-1544624-supplementary.pdf]

biosensors

Supplemental Information

Microsphere Peptide-Based Immunoassay for the Detection of Recombinant Bovine Somatotropin in Injection Preparations

1. Introduction

To develop a peptide microsphere-based immunoassay, peptides were coupled to the carboxyl-rich surface of the microspheres. In the literature, peptides are often coupled to microspheres using a biotin-streptavidin coupling [1–4], a spacer [5] or BSA conjugate [6,7]. Direct coupling of peptides to the microspheres is most straightforward; however, this is not common practice. Only a few articles describe directly coupled peptides in peptide-based microsphere immunoassays using a standard EDC/NHS protocol. These couplings are executed at pH 5 [8–10] and pH 7 [11,12]. However, when only the N-terminus is available to couple to the carboxylated microspheres, the pH should be adjusted in order to deprotonate the free aminogroup (pK_a ~9). To the best of our knowledge, peptide coupling at high pH has not been described for microsphere-based immunoassays before; however, optimized protocols for binding of small negatively charged molecules are known from SPR where high salt and pH are used to establish binding [13], and this approach has been executed in our study.

2. Results

2.1 Evidence of Peptides on the Microspheres

As mentioned in the manuscript, as expected, no binding occurred with the scrambled peptide. To ensure that also the scrambled peptide was correctly coupled to the microspheres and that these microspheres were a true blank, three sets of microspheres were characterized in addition by X-ray photoelectron spectroscopy (XPS). Figure S1 shows an XPS narrow scan of C1s, N1s and S2p for modified and non-modified microspheres. The C1s spectrum of pep-K was fitted with four peaks as hydrocarbon (C−H/C−C) at 285.0 eV, heteroatoms-bound carbon (C−N and C−S) at 286.7 eV, carbonyl groups from amide (HN−C=O) at 288.3 eV and finally carboxylic acid carbonyl carbon (C−C=O) group at 290.6 eV. The ratio carbonyl groups from amide and carboxylic acid carbonyl carbon (HN−C=O)/(C−C=O) in pep-K modified microsphere is 2.03 in accordance with the theoretically (amide carbonyl 11 and acid carbonyl 6) expected ratio of 2.2. In addition, the pep-K modified microsphere shows the characteristic ammonium (402 eV) and amide nitrogen (400 eV) in the N1s narrow scan spectra, and the S2p peak appearing at 164.7 eV, confirming the successful peptide coupling on the microspheres. The C1s spectrum of the pep-scr was fitted with four peaks as hydrocarbon (C−H/C−C) at 285.0 eV, heteroatom-bound carbon (C−N and C−S) at 286.7 eV, carbonyl groups from amide (HN−C=O) at 288.6 eV and finally carboxylic acid carbonyl carbon (C=O) group at 290.7 eV. The ratio carbonyl groups from amide and carboxylic acid carbonyl carbon (HN−C=O)/(C−C=O) in scr-pep modified microsphere is 2.6, in accordance with the theoretically (amide carbonyl 10 and acid carbonyl 4) expected ratio of 2.5. In addition, the pep-scr-modified microsphere showed the characteristic ammonium (402 eV) and amide nitrogen (400 eV) in the N1s narrow scan spectra, and S2p peak appeared at 164.4 eV, which is again confirming the successful coupling of pep-scr on the microsphere.

2.2 Quantification of the Amount of Peptide Immobilized on the Microspheres

Quantification of the amount of peptide immobilized on the microspheres via the XPS technique is quite reliable. We have estimated the percentage of attachment of peptides by considering 1 N1s atom in unmodified beads, and after modification with pep-K (DLEEGILALMRK), it will be 17 N1s (16 nitrogen from pep-K). Using this, and the fact

that the peptide contains a sulfur in methionine, we were able to calculate the N1s:S2p ratio in order to determine the loading of the peptide on the beads. Specifically, the theoretical N1s:S2p ratio for pep-K attached on the microspheres should be 17.0:1.0 and the experimental obtained 17.8:1.0, suggesting that >95% of pep-K attached on the microspheres. In case of scrambled peptide MRIEGLADLEL (Pep-scr), the theoretical N1s:S2p ratio on the microspheres should be 15.0:1.0 and the experimental obtained 15.5:1.0. Here, considering 1 N1s groups in unmodified beads and after modification with Pep-scr, it will be 15 N1s (14 nitrogen form Pep-scr) and 1 S2p, suggesting that >96% pep-scr attached on the microspheres.

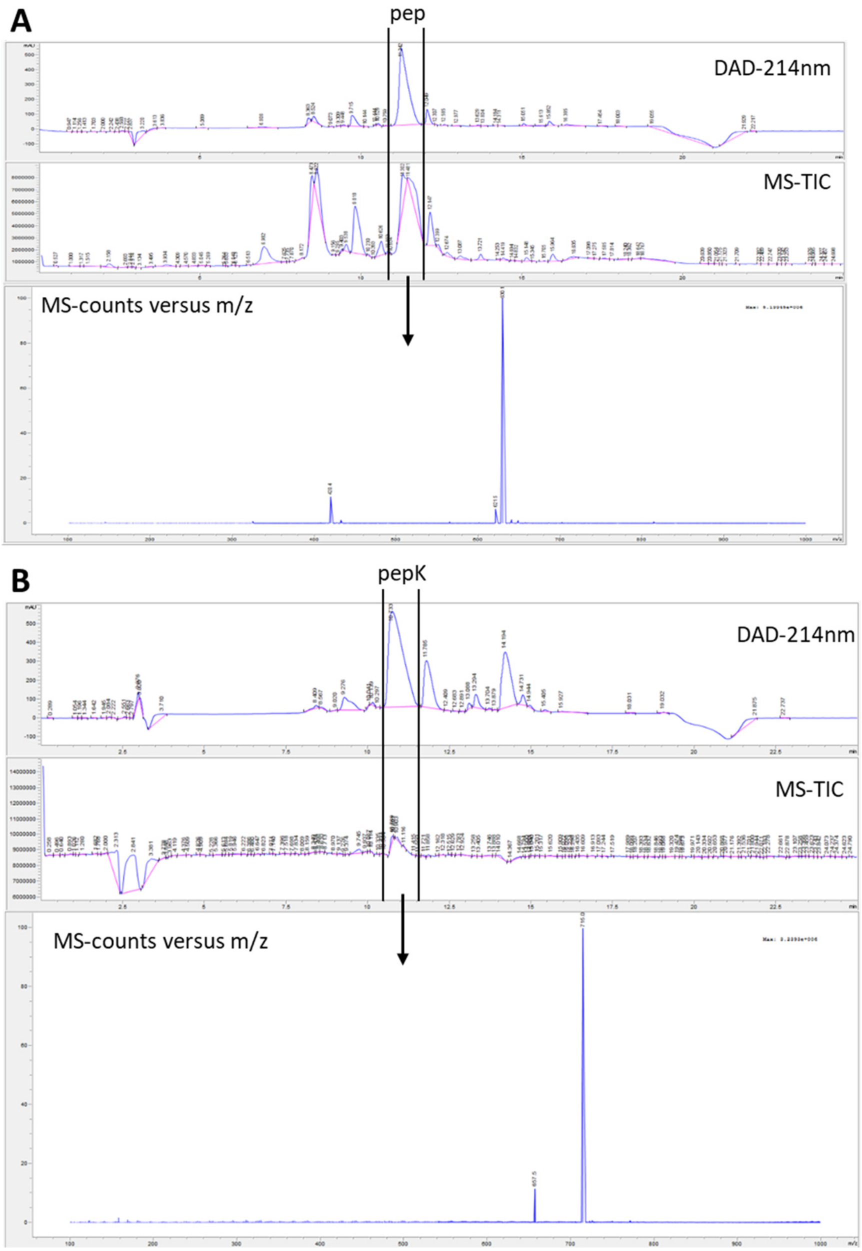

C

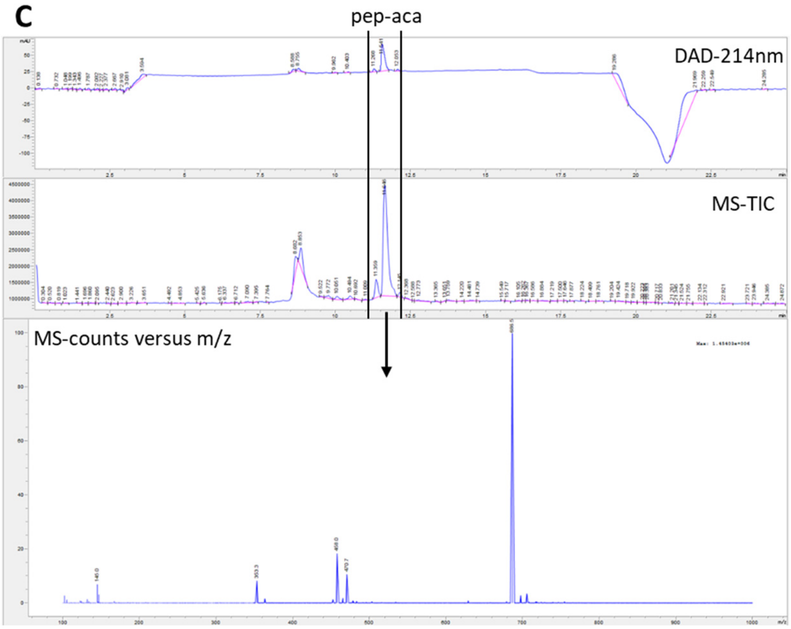

D

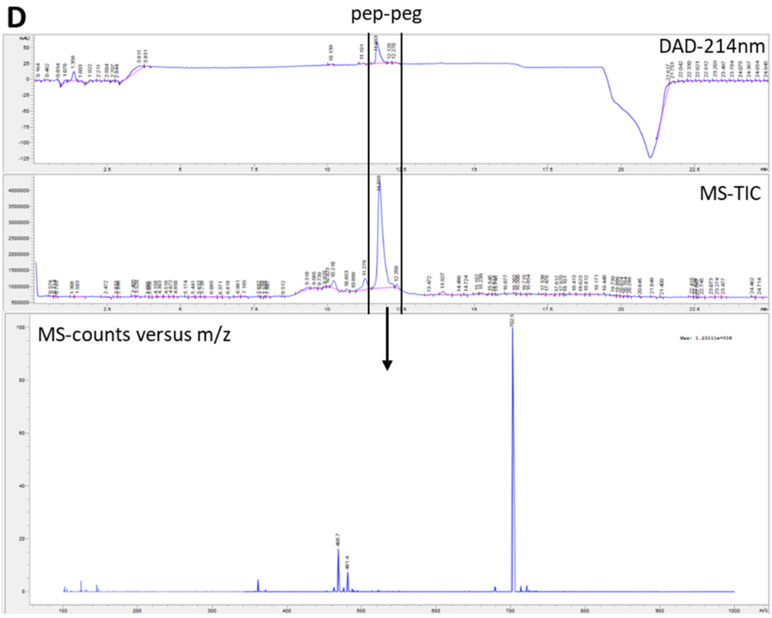

Figure S1. HPLC-DAD (diode array detection) chromatograms (top) and corresponding MS-total ion current (TIC) chromatograms (middle) for **A**) pep, **B**) pepK, **C**) pep-aca and **D**) pep-peg. Collected fraction is stated between the horizontal lines and the mass charge versus total counts of the fraction is shown (bottom).

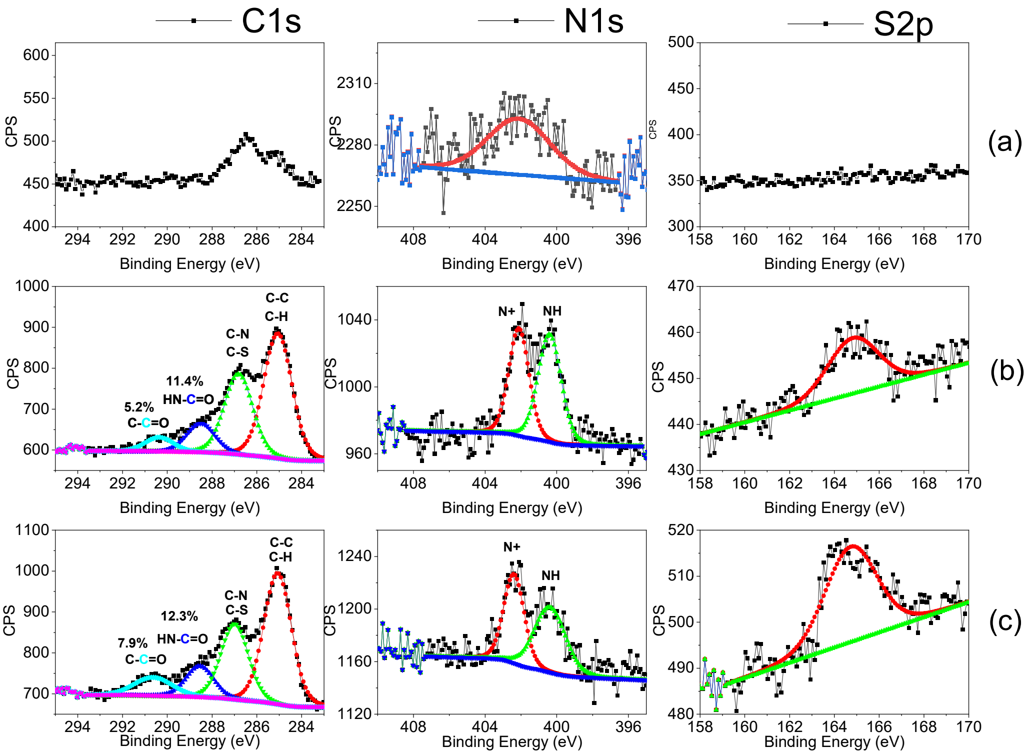

Figure S2. Narrow scan XPS spectra of C1s, N1s and S2p for microspheres: (**a**) non-modified microsphere-blank, (**b**) microsphere coupled with peptide DLEEGILALMRK and (**c**) microsphere coupled with scrambled peptide MRIEGLADLEL.

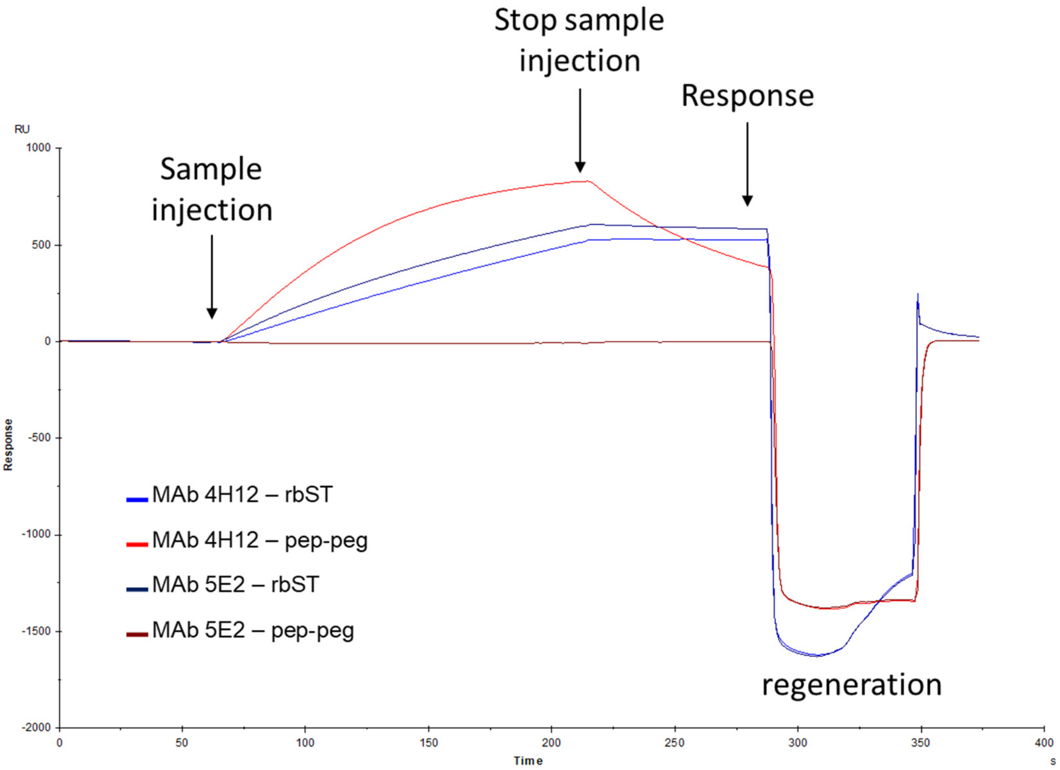

Figure S3. Surface plasmon resonance analysis to show binding of MAb 4H12 and MAb 5E2 on a flow channel (Fc) immobilized with rbST and an Fc immobilized with pep-peg.

Table S1. Response Units (RU) of surface plasmon resonance measurements. Binding of two anti-rbST MAbs were tested (Mab 4H12 and MAb 5E2) for rBST binding and Pep-peg binding. With the latter being the specific epitope for Mab 4H12.

**Mab**	**rbST (RU)**	**Pep-peg (RU)**
4H12	530	408
5E2	584	0

Table S2. Mean fluorescence intensities (MFI) of Mab 4H12 final dilutions (fd) on microspheres coupled with pep, pep-K, pep-aca, pep-peg and rbST.

**Mab 4H12 conc. (ng mL^{-1})**			**Microsphere**		
	**Pep (MFI)**	**Pep-K (MFI)**	**Pep-aca (MFI)**	**Pep-peg (MFI)**	**rbST (MFI)**
100	5807	14096	14535	20057	23988
50	3953	11471	11777	17707	22331
25	2234	8137	8917	14113	18510
12.5	1277	5629	5581	10443	14056
6.25	711	3413	3146	6949	9384
3.13	323	1805	1630	4171	5645
1.56	170	939	885	2220	3428
0.78	97	466	410	1170	1985

References

1. Hotop, S.-K., Abd El Wahed A., Beutling, U., Czerny, F., Sievers, C., Diederichsen, U., Frank, R., Stahl-Hennig, C., Brönstrup, M., Fritz, H.-J. Serological Analysis of Herpes B Virus at Individual Epitope Resolution: From Two-Dimensional Peptide Arrays to Multiplex Bead Flow Assays. *Anal. Chem.* **2019**, 91(17), 11030-11037.
2. Liang, B., Ge, C., Lönnblom, E., Lin, X., Feng, H., Xiao, L., Bai, J. A., Ayoglu, B., Nilsson, P., Nandakumar, K. S., Zhao, M., Holmdahl, R. The autoantibody response to cyclic citrullinated collagen type II peptides in rheumatoid arthritis. *Rheumatology* **2019**, 58(9), 1623-1633.
3. Sackesen, C., Suárez-Fariñas, M., Silva, R., Lin, J., Schmidt, S., Getts, R., Gimenez, G., Yilmaz, E. A, Cavkaytar, O., Buyuktiryaki, B., Soyer, O., Grishina, G., Sampson, H. A. A new Luminex–based peptide assay to identify reactivity to baked, fermented, and whole milk. *Allergy* **2019**, 74(2),327-336.
4. Suprun, M., R. Getts, R. Raghunathan, G. Grishina, M. Witmer, G. Gimenez, H. A. Sampson, and M. Suárez-Fariñas. Novel bead-based epitope assay is a sensitive and reliable tool for profiling epitope-specific antibody repertoire in food allergy. *Scientific Reports* **2019**, 9(1):18425.
5. Wilkerson, M. J., Black, K. E., Lanza-Perea, M., Sharma, B., Gibson, K., Stone, D. M., George, A., Nair, A. D., Ganta, R. R. Initial development and preliminary evaluation of a multiplex bead assay to detect antibodies to Ehrlichia canis, Anaplasma platys, and Ehrlichia chaffeensis outer membrane peptides in naturally infected dogs from Grenada, West Indies. *J. Vet. Diagn. Invest.* **2017**, 29(1), 109-114.
6. Kerkhof, K., Canier, L., Kim, S., Heng, S., Sochantha, T., Sovannaroth, S., Vigan-Womas, I., Coosemans, M., Sluydts, V., Ménard D. Implementation and application of a multiplex assay to detect malaria-specific antibodies: a promising tool for assessing malaria transmission in Southeast Asian pre-elimination areas. *Malar. J.* **2015**, 14(1), 1-14.
7. Koffi, D., Varela, M.-L., Loucoubar, C., Beourou, S., Vigan-Womas, I., Touré, A., Djaman, J. A., Touré, A. O., Perraut, R. Longitudinal analysis of antibody responses in symptomatic malaria cases do not mirror parasite transmission in peri-urban area of Côte d'Ivoire between 2010 and 2013. *PloS ONE* **2017**, 12(2), e0172899.
8. Fonseca, A.M., Quinto, L., Jiménez, A., González, R., Bardají, A., Maculuve, S., Dobaño, C., Rupérez, M., Vala, A., Aponte, J.J., Sevene, E., Macete, E., Menéndez, C., Mayor, A. Multiplexing detection of IgG against Plasmodium falciparum pregnancy-specific antigens. *PLoS ONE* **2017**, 12(7), e0181150.
9. Matsueda, S., Komatsu, N., Kusumoto, K., Koga, S., Yamada, A., Kuromatsu, R.,S., Yamada R., Seki R., Yutani S., Shichijo, S. Humoral immune responses to CTL epitope peptides from tumor-associated antigens are widely detectable in humans: a new biomarker for overall survival of patients with malignant diseases. *Dev. Comp. Immunol.* **2013**, 41(1), 68-76.
10. Yufenyuy, E.L., Parekh B.S. Development of a multiplex assay for concurrent diagnoses and detection of HIV-1, HIV-2, and recent HIV-1 infection in a single test. *AIDS Res. Hum. Retroviruses* **2018**, 34(12), 1017-1027.
11. Embers, M.E., Hasenkampf, N.R., Barnes, M.B., Didier, E.S., Philipp M.T., Tardo, A.C. Five-antigen fluorescent bead-based assay for diagnosis of lyme disease. *Clin. Vaccine Immunol.* **2016**, 23(4), 294-303.

12. Taitt, C.R., Shriver-Lake L.C., Anderson G.P., Ligler F.S. Surface modification and biomolecule immobilization on polymer spheres for biosensing applications, in Biomed. Nanotechnol.; Hurst S.J. (ed.); Humana Press. 2011, 726, 77-94.
13. Fischer, M. J. E. 2010. Amine Coupling Through EDC/NHS: A Practical Approach, in: Surface Plasmon Resonance: Methods and Protocols; Mol, N. J., Fischer, M. J. E. (Eds.); Humana Press, Totowa, New Jersey. pp 55-73
